# Antitumour effects of artesunate via cell cycle checkpoint controls in human oesophageal squamous carcinoma cells

**DOI:** 10.1186/s40001-024-01882-9

**Published:** 2024-05-22

**Authors:** Linlin Mao, Guodong Deng, Mengfan Li, Shih-Hsin Lu, Wei Jiang, Xiying Yu

**Affiliations:** 1 Department of Etiology and Carcinogenesis, National Cancer Center/National Clinical Research Center for Cancer/Cancer Hospital, Chinese Academy of Medical Sciences and Peking Union Medical College, Beijing, 100021 China; 2 State Key Laboratory of Molecular Oncology, National Cancer Center/National Clinical Research Center for Cancer/Cancer Hospital, Chinese Academy of Medical Sciences and Peking Union Medical College, Beijing, 100021 China; 3 Beijing Key Laboratory for Carcinogenesis and Cancer Prevention, National Cancer Center/National Clinical Research Center for Cancer/Cancer Hospital, Chinese Academy of Medical Sciences and Peking Union Medical College, Beijing, 100021 China; 4 Present Address: Affiliated Hangzhou First People’s Hospital, School of Medicine, Westlake University, Hangzhou, 310006 China

**Keywords:** Artesunate, DNA damage and cell cycle checkpoint controls, Oesophageal squamous cell carcinoma, Reactive oxygen species

## Abstract

Artesunate (ART), an effective antimalarial semisynthetic derivative of artemisinin, exhibits antitumour properties, but the mechanism(s) involved remain elusive. In this study, we investigated the antitumour effects of ART on human oesophageal squamous cell carcinoma (ESCC) cell lines. Treatment of ESCC cell lines with ART resulted in the production of excessive reactive oxygen species (ROS) that induced DNA damage, reduced cell proliferation and inhibited clonogenicity via G1-S cell cycle arrest and/or apoptosis in vitro. The administration of ART to nude mice with ESCC cell xenografts inhibited tumour formation in vivo. However, the cytotoxicity of ART strongly differed among the ESCC cell lines tested. Transcriptomic profiling revealed that although the expression of large numbers of genes in ESCC cell lines was affected by ART treatment, these genes could be functionally clustered into pathways involved in regulating cell cycle progression, DNA metabolism and apoptosis. We revealed that p53 and Cdk4/6-p16-Rb cell cycle checkpoint controls were critical determinants required for mediating ART cytotoxicity in ESCC cell lines. Specifically, KYSE30 cells with *p53*^*Mut*^*/p16*^*Mut*^ were the most sensitive to ART, KYSE150 and KYSE180 cells with *p53*^*Mut*^*/p16*^*Nor*^ exhibited intermediate responses to ART, and Eca109 cells with *p53*^*Nor*^*/p16*^*Nor*^ exhibited the most resistance to ATR. Consistently, perturbation of p53 expression using RNA interference (RNAi) and/or Cdk4/6 activity using the inhibitor palbociclib altered ART cytotoxicity in KYSE30 cells. Given that the p53 and Cdk4/6-cyclin D1-p16-Rb genes are commonly mutated in ESCC, our results potentially shed new light on neoadjuvant chemotherapy strategies for ESCC.

**Supplementary Information:**

The online version contains supplementary material available at 10.1186/s40001-024-01882-9.

## Background

Oesophageal cancer (EC) is one of the most fatal malignant tumours of the digestive system and has two main histological subtypes: oesophageal adenocarcinoma (EAC) and oesophageal squamous cell carcinoma (ESCC) [4]. Geographic variations in the incidence of EC are striking, with high incidences of ESCC in Eastern Asia and high incidences of EAC in Western countries [4, 15, 33]. In contrast to EAC, ESCC is often diagnosed during its advanced stages due to the lack of typical early clinical symptoms. Although multimodal therapy, such as combined chemoradiotherapy followed by surgery, has been recommended for locally advanced ESCC, the 5-year survival rate of patients with ESCC is poor, at only 20.9% in China [7, 15]. Recently, high-throughput omic technologies have been widely applied to explore the carcinogenesis of ESCC for the determination of driver genes and/or possible molecular targets. A large body of evidence reveals that ESCCs have high mutation burdens and heterogeneity, as identified by cell cycle checkpoint control-related genes, such as *TP53* (p53), *Cyclin D1*, *Cdk4/6*, *CDKN2A* (p16) and *Rb*, which are commonly mutated [3]. In addition, other important ESCC driver genes involved in regulating proliferation, differentiation, DNA metabolism and antioxidation-related biological events have been detected [13, 24, 36]. However, the complex genetic backgrounds of ESCC have resulted in dismal research progress in precision medicine. Better and more economical chemotherapeutic drugs or adjuvant drugs for treating ESCC are still needed to improve treatment efficacy.

Artemisinin, a sesquiterpene lactone isolated from the Chinese medicinal plant Sweet Wormwood (*Artemisia annua L., Asteraceae*), is an effective antimalarial drug [35]. When artemisinin interacts with haem and/or iron, the endoperoxide moiety of artemisinin is cleaved to generate reactive oxygen species (ROS) that exert toxic effects on *Plasmodium* [34, 37]. Thus, the endoperoxide moiety inside artemisinin is crucial for its biological effects. Because of the strong induction of ROS by artemisinin, numerous studies have suggested that the activity of artemisinin may not be restricted to antimalarial agents. Artemisinin could be used as a therapeutic agent for neoplasms, autoimmune disorders and other diseases [9, 14]. Indeed, many researchers have explored the effect(s) and possible mechanism(s) of artemisinin and its derivatives (dihydroartemisinin, artesunate, artemether, arteether, etc.) on cancer cells over the past two decades. In addition to these suppressive effects, the oxidative stress response induced by artemisinin-dependent ROS has remained the most broadly reported [2, 10, 23, 25]. In cancer cells, an ingenious balance between oxidants and antioxidants is required for viability and a malignant phenotype. Decreased intrinsic antioxidant capacity and/or exogenous agents promoting oxidation destroy redox equilibrium, causing genome instability, lipid peroxidation, aberrant protein production, endoplasmic reticulum stress, mitochondrial dysfunction and ultimately cancer cell death [26, 30]. It is postulated that ROS-producing agents are proper cancer treatments. However, other effects, including DNA damage and repair [2, 9, 22], inhibition of angiogenesis [1, 5, 38] and alteration of signal transduction pathways (e.g., the Wnt/β-catenin pathway, the AMPK pathway, and metastatic pathways) [19, 21, 28], have also been reported to be related to the generation of ROS in response to artemisinin treatment. In this study, we focused on investigating and determining the antitumour effects of ART in human ESCC cells.

## Materials and methods

### Cell lines and cell culture

The human ESCC cell lines KYSE150, KYSE180, KYSE30, and KYSE510 were gifts from Dr. Y. Shimada at Hyogo College of Medicine. TE1 and Eca109 cells were purchased from the Shanghai Cell Bank, Chinese Academy of Sciences. The identities of the cell lines were confirmed by standard Short Tandem Repeat (STR) analysis with reference to the American Tissue Culture Collection (ATCC). All cells were maintained in RPMI 1640 culture medium supplemented with 10% foetal bovine serum at 37 °C and 5% CO_2_. ART (Macklin Biochemical, China) was dissolved in dimethyl sulfoxide (DMSO) (10^4^ μM) as a stock solution and stored at 4 °C in aliquots until use. N-acetylcysteine (NAC; Beyotime, China) was used at a concentration of 10 mM 1 h before ART treatment. Palbociclib (PD0332991, Selleck Chemicals) was used at a concentration of 10 μM according to Chen et al. [6]. *TP53*-specific siRNAs, including 3 siRNAs and 1 negative control, were obtained from GenePharma. The siRNA sequences used were as follows: siRNA1 sense, 5'-CCGGACGAUAUUGAACAAUTT-3'; siRNA1 antisense, 5'-AUUGUUCAAUAUCGUCCGGTT-3'; siRNA2 sense, 5'-GUACCACCAUCCACUACAATT-3'; siRNA2 antisense; 5'-UUGUAGUGGAUGGUGGUACTT-3'; siRNA3 sense, 5'-GUAAUCUACUGGGACGGAATT-3'; siRNA3 antisense, 5'-UUCCGUCCCAGUAGAUUACTT-3'. siRNAs were transfected into KYSE30 cells with Lipofectamine 3000 from Invitrogen for 48 h. Transfection efficiency was validated by western blotting. The most effective siRNA was selected for transfection for 24 h, and the cells were used for further experiments. Mutation data for KYSE30, KYSE150, KYSE180, KYSE30, KYSE510 and TE1 cells were obtained from the Cancer Cell Line Encyclopedia website (https://portals.broadinstitute.org/ccle), whereas data for Eca109 cells were obtained from whole-genome sequencing in our laboratory (data not shown here). Pathogenicity and relevant studies of *TP53* mutations are available at the National Center for Biotechnology Information website (https://www.ncbi.nlm.nih.gov/clinvar or https://www.ncbi.nlm.nih.gov/snp).

### Xenografts assay in mice

Female BALB/c nude mice (SPF, 3–4 weeks old) were purchased from Vital River Laboratory Animal Technology Co., Ltd. (Beijing, China). Animals were kept in microisolator cages according to the guidelines of CAMS and PUMC, and all experiments were approved by the animal care committee of CAMS and PUMC. A total of 1.0 × 10^6^ KYSE150 cells were subcutaneously inoculated into the right flanks of the nude mice. Body weights and tumour volumes were measured every 3 days for 4 weeks. At the end of the study, the mice were killed, and the tumours were removed and weighed. L × W^2^/2 was used to determine the tumour volume (L indicates the length, and W indicates the width).

### Measurement of ROS levels

ROS production was detected using ROS-dependent oxidation of nonfluorescent 2′,7′-dichlorofluorescin diacetate (DCFH-DA, Beyotime, China) into the highly fluorescent compound 2′,7′-dichlorofluorescein (DCF). Cells were treated with vehicle, ART or NAC + ART for 12 h and then incubated with DCFH-DA (10 μmol/L) for 30 min at 37 °C. ROS levels were measured using flow cytometry (Becton Dickinson FACS Canto II, NJ, USA) or fluorescence microscopy (Leica, Germany).

### Cell viability and colony formation assays

The CCK-8 (Dojindo, Japan) assay was used to evaluate the viability of the six ESCC cell lines. The cells were seeded in a 96-well plate. After 24 h, various ART concentrations were added to each well through exchange of the medium. CCK-8 solution was added to each well at the indicated time points, and the cells were incubated for the appropriate time periods before the optical density (OD) was measured at 450 nm using a microplate spectrophotometer (pectraMax190, Molecular Devices, Sunnyvale, CA, USA). KYSE150 and KYSE180 cells were treated with various concentrations of ART for 24 h or 48 h and then plated in six-well plates at a density of 400 cells/well. After 14 days, the resulting colonies were fixed with methanol for 15 min and stained with 0.1% crystal violet for visualization and counting.

### FACS and apoptosis analyses

Cell cycle analysis was performed using a cell cycle detection kit (Beyotime, China). To assess the cell cycle, we collected control and treated cells, washed them with PBS and then fixed them with chilled 70% ethanol for 24 h at 4 °C. We then washed the fixed cells with PBS, incubated them with RNase A for 30 min, and stained them with propidium iodide for 30 min. Then, the samples were analysed using flow cytometry (Becton Dickinson FACS Canto II, NJ, USA). FlowJo X was used to determine the percentage of cells in each cell cycle phase.

Cell apoptosis was assessed using an Annexin V-FITC Apoptosis Detection Kit (Beyotime, China) according to the manufacturer’s instructions.

### Western blotting analysis

The cell lysates were separated using SDS‒PAGE and then transferred to polyvinylidene fluoride (PVDF) membranes (Millipore, USA), which were blocked with 5% BSA for 1 h at room temperature. The PVDF membranes were incubated with primary antibody (1:1000 dilution) overnight at 4 °C, washed and incubated with secondary antibody (1:10000 dilution, ZSGB-BIO, China) for 2 h at room temperature. The immunoreactive bands were visualized with an enhanced chemiluminescence (ECL) kit (Beyotime, China) according to the manufacturer’s instructions. The primary antibodies used were purchased from CST (γ-H2AX, Rb, p-Rb^Ser780^, p-Rb^Ser795^, MCM2, MCM3, p-ATM, p-ATR, chk1, chk2, p-chk1, and p-chk2), Abcam (Bcl-2, Bax, and p-Rb^Thr821^) and Proteintech (cyclinD1, cyclinE1, cyclinB1, cyclinA2, CDK2, CDK4, CDK6, caspase-3, caspase-8, and caspase-9).

### Immunofluorescence, immunohistochemistry (IHC) and tumour histology

Xenografts were removed from each mouse, fixed in formalin and then embedded in paraffin. Histopathological changes were assessed in tissue sections from each group. We prepared 4-μm sections and stained them with haematoxylin and eosin (H&E) to examine their tissue and cellular structures using light microscopy (Leica, Germany). Tissue sections were deparaffinized and hydrated. Endogenous peroxidase activity was inhibited by incubating the sections in 3% H_2_O_2_. After antigen retrieval and nonspecific reaction blocking, the sections were incubated with primary antibodies against caspase-3, -8, and -9 (1:300 dilution, Proteintech, China) at 4 °C overnight. The sections were then subjected to IHC with a specific kit (SP9002, ZSGB-BIO, China) according to the manufacturer’s instructions, followed by haematoxylin counterstaining. In each Section 6 nonrandomly selected fields were photographed at 100 × and 400 × magnification (Leica, Germany). The sections were then evaluated for positive signal intensity and percentage.

Cells grown on coverslips were treated with various concentrations of ART for 24 h and fixed with 4% formaldehyde, followed by treatment with 0.2% Triton X-100 in PBS for 5 min. Then, the cells were blocked with 5% bovine serum albumin in PBS containing 0.3% Triton X-100 for 30 min. The specimens were incubated with primary γ-H2AX antibody (1:200, CST) overnight at 4 °C. After washing, the specimens were incubated with a rhodamine-labelled secondary antibody (1:200, ZSGB-BIO, China) for 60 min in the dark and then counterstained with 4,6-diamidino-2-phenylindole for 5 min. Antifade solution was used to mount the specimens on the slides. The slides were then examined under a laser scanning confocal microscope (LSCM, Leica, Germany). At least 500 nuclei were scored for nuclear foci in each of 3 experimental repeats.

### Transcriptomic (RNA-seq) analysis

RNA was extracted from KYSE150 and KYSE180 cells using TRIzol (Beyotime, China). The extracted RNA samples were first examined for concentration and purity to exclude degradation or contamination using a Qubit and Nanodrop spectrophotometer. For library construction, nonstranded cDNA libraries were constructed using poly(A) mRNA enrichment and the NEBNext UltraTM II RNA Library Prep Kit for Illumina following the manufacturer’s instructions. RNA and library qualities were confirmed using fragment analysis (Agilent 2100 Bioanalyzer). FastQC software (version 0.11.7) was used for quality control of the raw data, and Illumina was used to evaluate the sequencing error rate and base quality. For gene differential expression analysis, EBSeq was used to obtain the differentially expressed gene sets between the two samples, and fold change ≥ 2 or ≤ 1/2 and FDR < 0.01 were used as the screening standards. To investigate differences in the biological processes associated with these DEGs, Gene Ontology (GO) enrichment was performed (*Q* ≤ 0.05 was considered to indicate significant enrichment). Next, the enriched annotated genes were subjected to Kyoto Encyclopedia of Genes and Genomes (KEGG) functional analysis.

### Statistical analysis

All the data were analysed using GraphPad Prism 7.0 and SPSS 22.0 software. The data are expressed as the mean ± SD, and the data from specific experiments were compared using one-way ANOVA or Student’s t test. *P* < 0.05 was considered to indicate statistical significance.

## Results

### ART induced ROS, DNA damage and cell death in ESCC cells

**Fig. 1:**
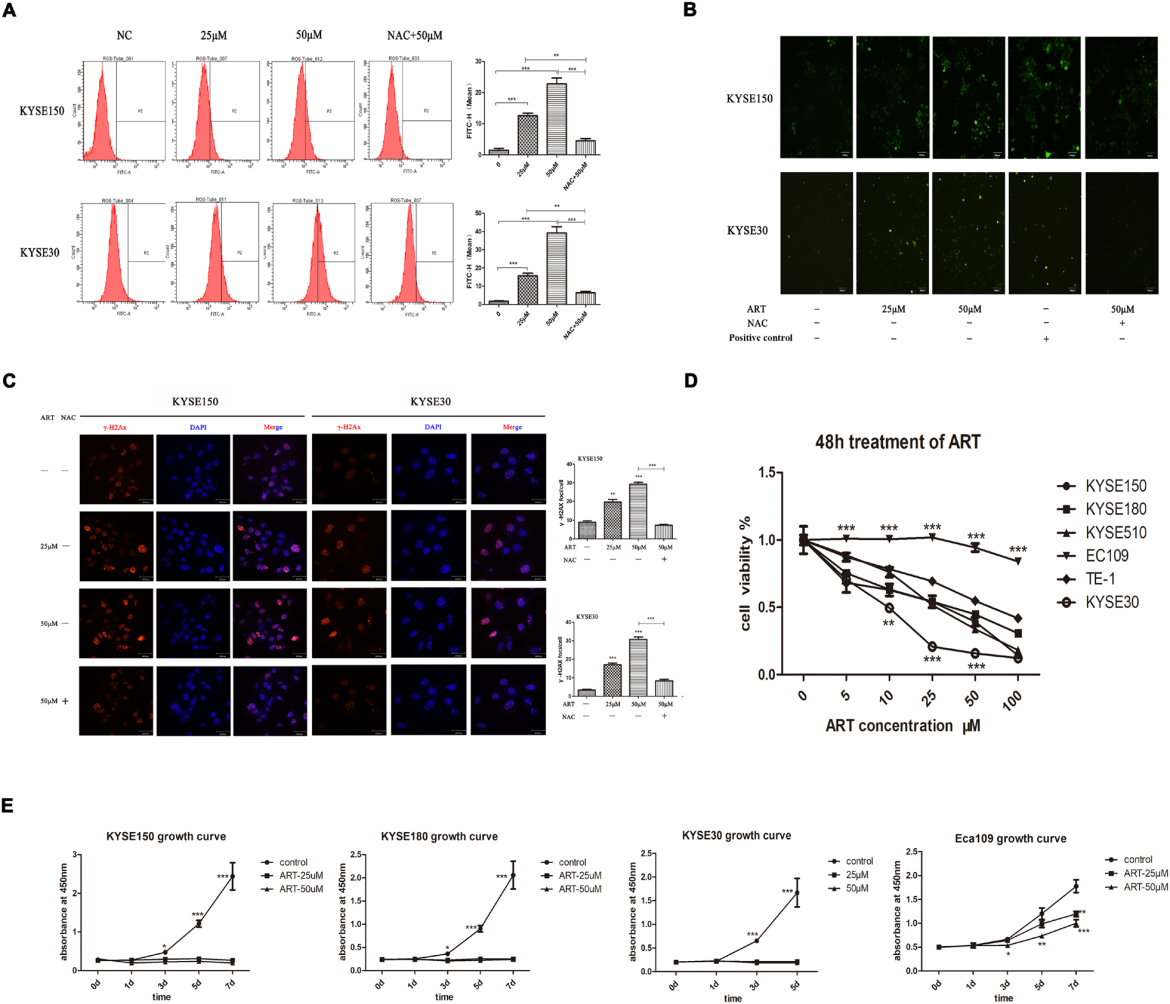
Intracellular ROS changes, DNA damage and cell viability in ESCC cells treated with ART. **A** Intracellular ROS changes induced by different concentrations of ART and the inhibition of ROS generation by NAC were detected using flow cytometry in KYSE150 and KYSE30 cells. **B** Intracellular ROS changes induced by different concentrations of ART and the inhibition of ROS generation by NAC were assessed using fluorescence microscopy in KYSE150 and KYSE30 cells. **C** Immunofluorescence staining of KYSE150 and KYSE30 cells treated with different ART concentrations for 24 h and NAC-pretreated cells. Nuclei were stained blue with DAPI; γ-H2AX foci appear as red fluorescent dots on the blue background. Original magnification: 400x. **D** The effects of different concentrations of ART on the viability of ESCC cell lines at 48 h, as determined using the CCK-8 assay. **E** The effects of different ART concentrations on KYSE150, KYSE180, KYSE30 and Eca109 cell proliferation determined using the CCK-8 assay. *n* = 6, *P < 0.05, **P < 0.01, ***P < 0.001

Previous studies have demonstrated that ART has antitumour effects on ESCC cells. Shi et al. showed that ART treatment significantly suppressed KYSE150 cell proliferation [29]. Jin et al. reported that artesunate had an anti-EC effect by inhibiting aerobic glycolysis in both KYSE150 and KYSE170 cells [16]. In addition, studies have indicated that the proliferation of ESCC cells, such as KYSE150, KYSE410, and TE-1 cells, is inhibited 48 h after ART treatment in a dose-dependent manner [12]. Therefore, we investigated the effects of ART in two ESCC cell lines, KYSE30 and KYSE150, which were treated with ART in vitro. We first determined whether ART induced ROS production in these ESCC cells. Intracellular ROS were monitored in KYSE30 and KYSE150 cells exposed to different concentrations of ART for 12 h using FACS. As shown in Fig. 1A, B, ART treatment caused dose-dependent increases in ROS production in both ESCC cell lines. However, when KYSE30 and KYSE150 cells were pretreated with the antioxidant N-acetyl-L-cysteine (NAC) for 1 h before ART treatment, ART-dependent ROS production was greatly abrogated in these ESCC cells (Fig. 1B). These results indicated that ART strongly induces ROS in ESCC cells.

Given that high levels of ROS in cells can induce several cellular oxidative stress responses, including redox response and DNA damage response, we next examined whether ROS response markers, such as γ-H2AX foci formation (DNA damage response), were altered in ART-treated KYSE30 and KYSE150 cells. As shown in Fig. 1C, compared with controls, treatment with ART in KYSE30 and KYSE150 cells strongly induced γ-H2AX foci formation. Consistently, ART-induced γ-H2AX foci formation was dose dependent, whereas pretreatment of ESCC cells with NAC significantly reduced ART-induced γ-H2AX foci formation (Fig. 1C). Thus, ART induces ROS, resulting in excessive cellular oxidative stress, such as DNA damage, in ESCC cells.

The occurrence of excessive DNA damage caused by ART in ESCC cells prompted us to test whether ART treatment affects the viability of ESCC cells. To this end, a panel of ESCC cell lines (KYSE150, KYSE180, KYSE510, KYSE30, TE-1 and Eca109) treated with and without ART were examined using the CCK-8 assay. As shown in Fig. 1D, ART treatment reduced cell viability in a dose-dependent manner in all 6 ESCC cell lines. ART also exerted its pharmacological effect in a time-dependent manner in KYSE150 and KYSE180 cell lines (Fig. 1E). Interestingly, the cytotoxicity of ART strongly differed among the ESCC cell lines. KYSE30 cells showed the greatest sensitivity to ART, followed by KYSE150, KYSE180, KYSE510 and TE-1 cells, whereas Eca109 cells remained the most resistant. The half-maximal inhibitory concentration (IC50) values of ART in KYSE30, KYSE150, KYSE180, KYSE510, TE-1 and Eca109 cells were 5, 21, 30, 27, 66 and 213 μM, respectively. The IC50 of ART in the most resistant cell line, Eca109, was 40-fold greater than that in the most sensitive cell line, KYSE30, indicating that the ability of ART to kill ESCC cells was cell line dependent (Table 1).

**Table 1:** IC50s and genomic alterations ESCC cell lines

Cell lines	IC50	Genomic alteration
KYSE30	9.95 ± 0.56 μM	CDKN2A stop-gain mutation
KYSE150	20.86 ± 1.15 μM	TP53 mutation
KYSE180	30.27 ± 1.04 μM	TP53 mutation
KYSE510	27.36 ± 1.04 μM	TP53 mutation
TE-1	66.05 ± 1.08 μM	TP53 mutation
Eca109	213.80 ± 1.37 μM	TP53^+/+^, CDKN2A^+/+^

### ART-induced cell death was due to cell cycle arrest, ultimately triggering ESCC apoptosis

**Fig. 2:**
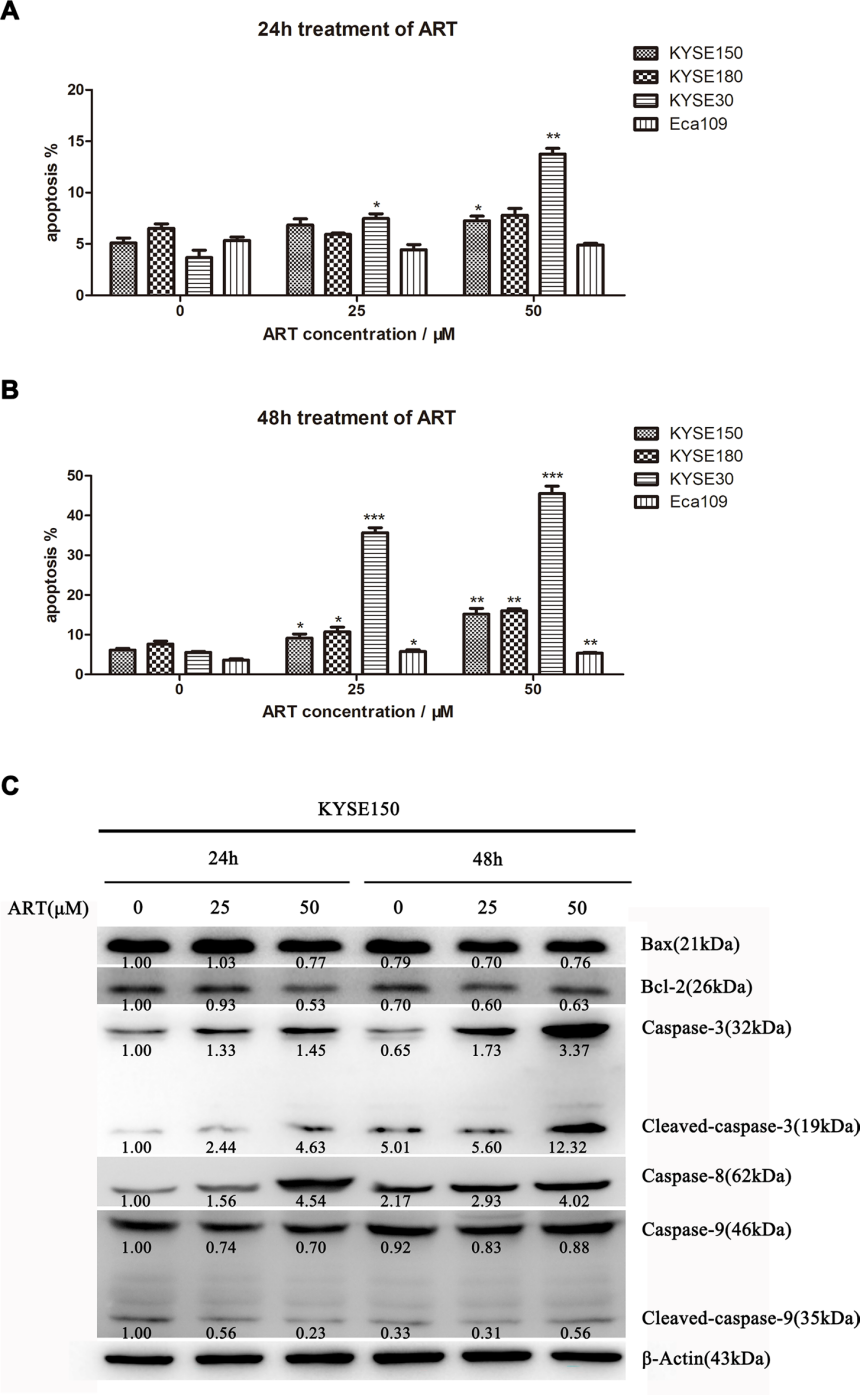
The effects of different ART concentrations on ESCC cell apoptosis. **A**–**B** KYSE150, KYSE180, KYSE30 and Eca109 cells were treated with 25 μM or 50 μM ART for 24 h or 48 h, and apoptotic cells were detected using flow cytometry. The percentages of apoptotic cells are the sum of early apoptotic cells and late apoptotic cells. *n* = 3, *P < 0.05, **P < 0.01, ***P < 0.001. **C** Western blot analyses were performed for apoptosis-related proteins (Bcl-2 family proteins and caspases) in KYSE150 cells treated with 25 μM or 50 μM ART for 24 or 48 h

To better understand how ART reduces cell viability and the possible underlying mechanism(s), we selected 4 ESCC cell lines—KYSE30 (an ART-sensitive cell line), KYSE150 and KYSE180 (ART-moderately sensitive cell lines) and Eca109 (an ART-resistant cell line)—for further studies. The cell cycle and apoptosis status of KYSE30, KYSE150, KYSE180, and Eca109 cells treated with or without ART were assessed using FACS and immunoblotting analyses. As shown in Fig. 2A, B and Additional file 1, ART exposure (25 μM or 50 μM) for 24 or 48 h in these four ESCC cell lines induced a significant concentration-dependent increase in cell death in the KYSE30, KYSE150 and KYSE180 cell lines but not in the Eca109 cell line. The mean cell death rates of KYSE30 cells were greater than those of KYSE150 and KYSE180 cells under the same ART treatment conditions. In contrast, Eca109 cells were resistant to ART treatment (25 μM or 50 μM), consistent with the results obtained from the cell viability analysis (Fig. 1D). To determine how ART kills ESCC cells, the expression of apoptosis regulatory proteins was examined using immunoblotting analyses. As shown in Fig. 2C, the expression of the proapoptotic proteins cleaved caspase-3, caspase-8 and Bax/Bcl-2 was significantly increased in ESCC cells exposed to ART. These results indicated that the cell death induced by ART in ESCC cells was due to caspase-dependent apoptosis.

**Fig. 3:**
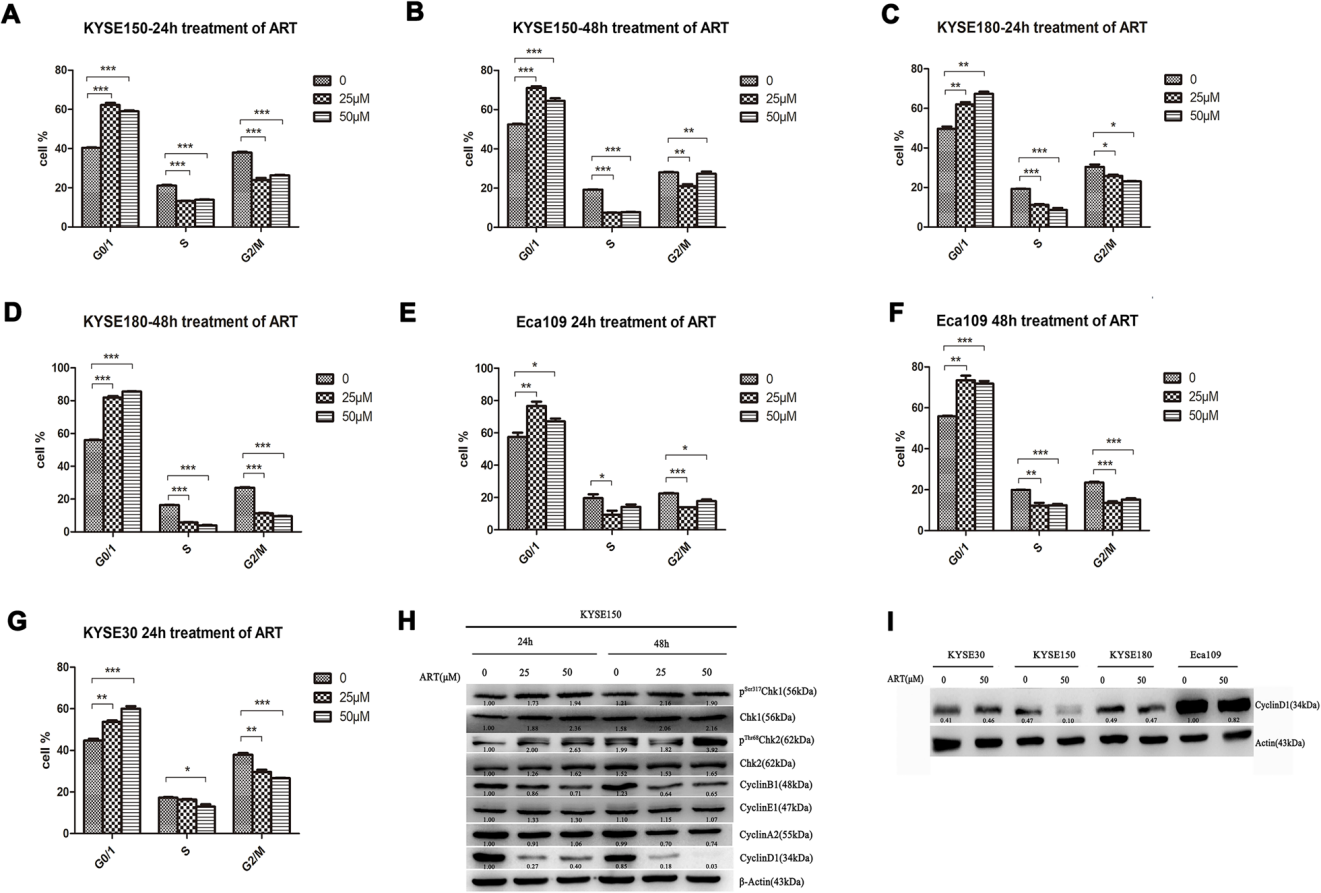
The effects of ART concentrations on the cell cycle in ESCC cells. **A**–**G** The proportions of estimated cell cycle phases in ESCC cell lines treated with different ART concentrations. Cells were treated with 25 μM or 50 μM ART for 24 h or 48 h, and DNA ploidy was assessed by propidium iodide (PI) staining and flow cytometry. *n* = 3, *P < 0.05, **P < 0.01, ***P < 0.001. **H** Western blot analyses were performed for cell cycle-related proteins in KYSE150 cells treated with 25 μM or 50 μM ART for 24 or 48 h. **I** Western blot analyses were performed for cyclin D1 in different ESCC cell lines after treatment with 50 μM ART for 24 h

We assessed how ART-sensitive ESCC cells undergo apoptosis and how ART-resistant ESCC cells do not die after ART treatment. The cell cycle progression profiles of the four ESCC cell lines treated with or without ART were monitored using FACS. As shown in Fig. 3A–G and Additional file 2, significant changes in cell cycle profiles were noted in all four ESCC cell lines after ART treatment. Compared with those of the controls, the ratio of cells in the S-G2/M phase gradually decreased, whereas the ratio of cells in the G0/G1 phase gradually increased in ART-treated ESCC cells in a dose- and time-dependent manner. However, compared with ART-resistant Eca109 cells, which only displayed an accumulation of cells in G1 arrest without an accumulation of cells in the sub-G1 phase after cells were treated with 25 μM or 50 μM ART for 24 or 48 h, treatment of moderately sensitive cell lines, KYSE150 and KYSE180, with ART yielded not only an increase in the G1 arrest profile, but also a significant increase in the sub-G1 cell death profile in a dose- and time-dependent manner. In contrast, an increase in the G1 arrest profile together with a large increase in the sub-G1 cell death profile was detected only in the ART-sensitive cell line KYSE30 treated with 25 μM or 50 μM ART for 24 h. After KYSE30 cells were treated with 25 μM or 50 μM ART for 48 h, most of the cells died. Taken together, these results indicated that ART initially induced cell cycle arrest in ESCC cells. Moreover, ART ultimately killed ART-sensitive ESCC cells by triggering apoptosis.

Consistently, immunoblotting analysis of the cell cycle/checkpoint-related proteins cyclin D1, cyclin E1, cyclin B1, cyclin A2, Chk1/phospho-Chk1 and Chk2/phosphor-Chk2 in KYSE150 cells demonstrated that cell cycle-related proteins were decreased but that the phosphorylation of DNA damage checkpoint proteins was increased in ART-treated cells compared with non-treated cells (Fig. 3H). Decreased cyclin D1 expression was detected in ART-treated cells, in line with the G1 cell cycle arrest of these cells observed using FACS (Fig. 3I).

### ART inhibited the clonogenicity and tumorigenic ability of ESCC cells in vitro and in vivo

**Fig. 4:**
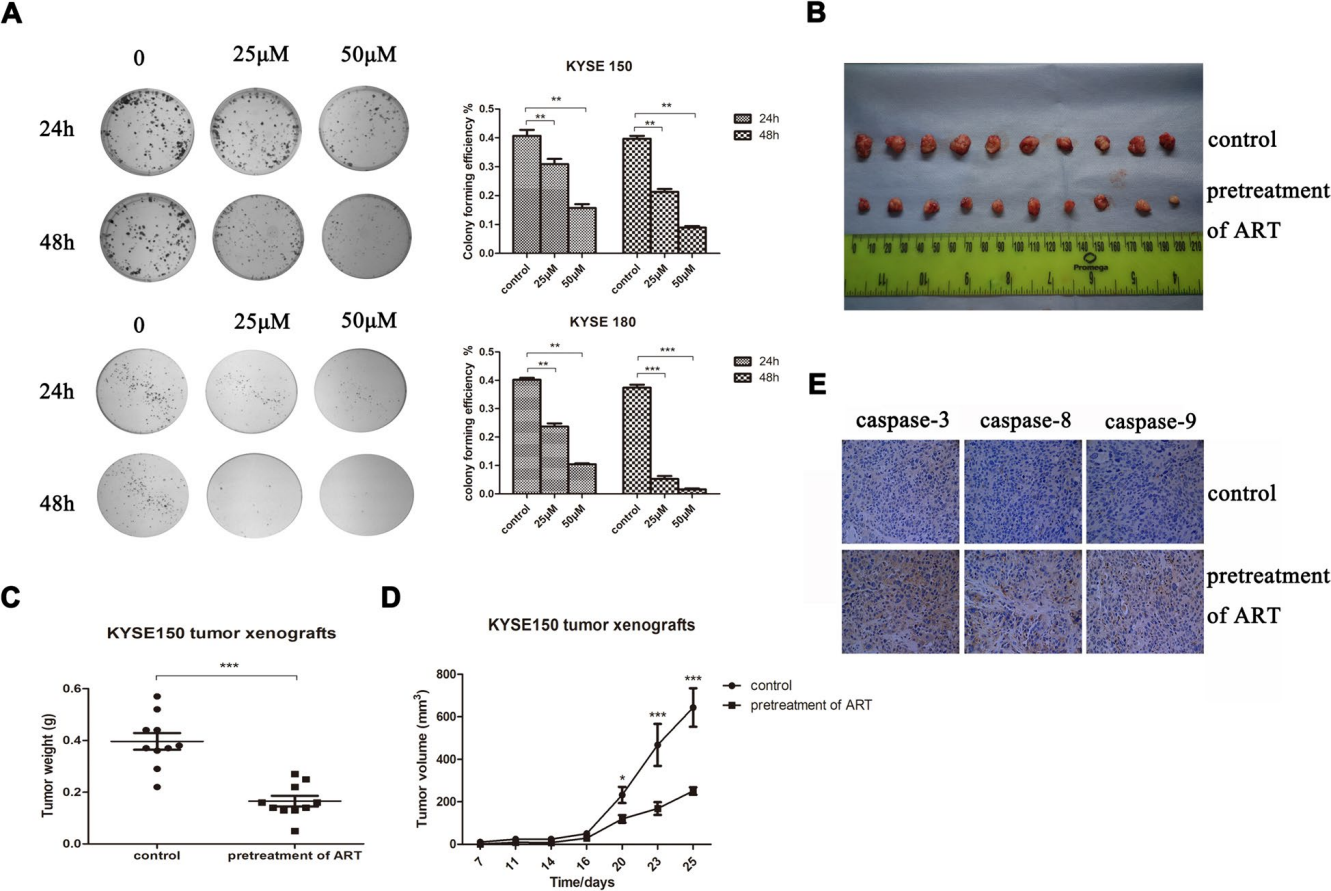
The effects of different concentrations of ART on the colony formation and tumorigenicity of ESCC cells. **A** The oesophageal cancer cell lines KYSE150 and KYSE180 were treated with 25 μM and 50 μM ART for 24 or 48 h and then harvested and plated in the control group for colony formation assays. **B** KYSE150 cells were used to establish xenograft tumours in BALB/c mice, and KYSE150 cells were pretreated with 50 μM ART for 48 h or vehicle (DMSO as a control) before subcutaneous injection. Then, 25 days later, representative xenograft tumours from ART-treated mice and vehicle-treated mice were generated. **C** Overall weight of the dissected tumours. *n* = 10. **D** Changes in the mean tumour volume in ART-treated mice and vehicle-treated mice. **E** Immunohistochemical staining of KYSE150 xenograft tumours for apoptosis markers (caspases) in oesophageal cancer cells. Original magnification: 400x. *P < 0.05, **P < 0.01, ***P < 0.001

To determine whether ART-induced cell cycle arrest and/or apoptosis in ESCC cells affects their carcinogenicity potential, we first performed a plate colony formation assay in vitro. KYSE150 and KYSE180 cells were treated with 0, 25 and 50 μM ART for 24 or 48 h before being seeded in 6-well plates. The colony formation ability of these cells was determined using a colony formation assay. As shown in Fig. 4A, ART treatment significantly decreased the colony formation efficiency of KYSE150 and KYSE180 cells. To further evaluate the effects of ART on the tumorigenic ability of ESCC cells in vivo, we examined the effects of ART on KYSE150 cell growth in mouse xenograft models. As shown in Fig. 4B–D, the administration of ART to nude mice inhibited the growth of KYSE150-transplanted tumours. The volume and weight of the transplanted tumours in the ART-treated group were lower than those in the control group (*P* < 0.05). We assessed the expression of apoptosis-related proteins (caspase-3, caspase-8, and caspase-9) in tumours obtained from nude mice treated with or without ART using immunohistochemistry. As shown in Fig. 4E, the levels of these proteins were greater in tumours obtained from ART-treated nude mice than in those obtained from control mice, similar to the results obtained from cultured ESCC cells in vitro (Fig. 2C). Hence, these results indicated that ART not only blocked ESCC cell proliferation via cell cycle arrest/apoptosis in vitro, but also inhibited ESCC cell tumorigenicity in vivo.

### ART induced cell cycle arrest/apoptosis via p53 and Cdk4/6-p16-Rb checkpoint controls in ESCC cells

**Fig. 5:**
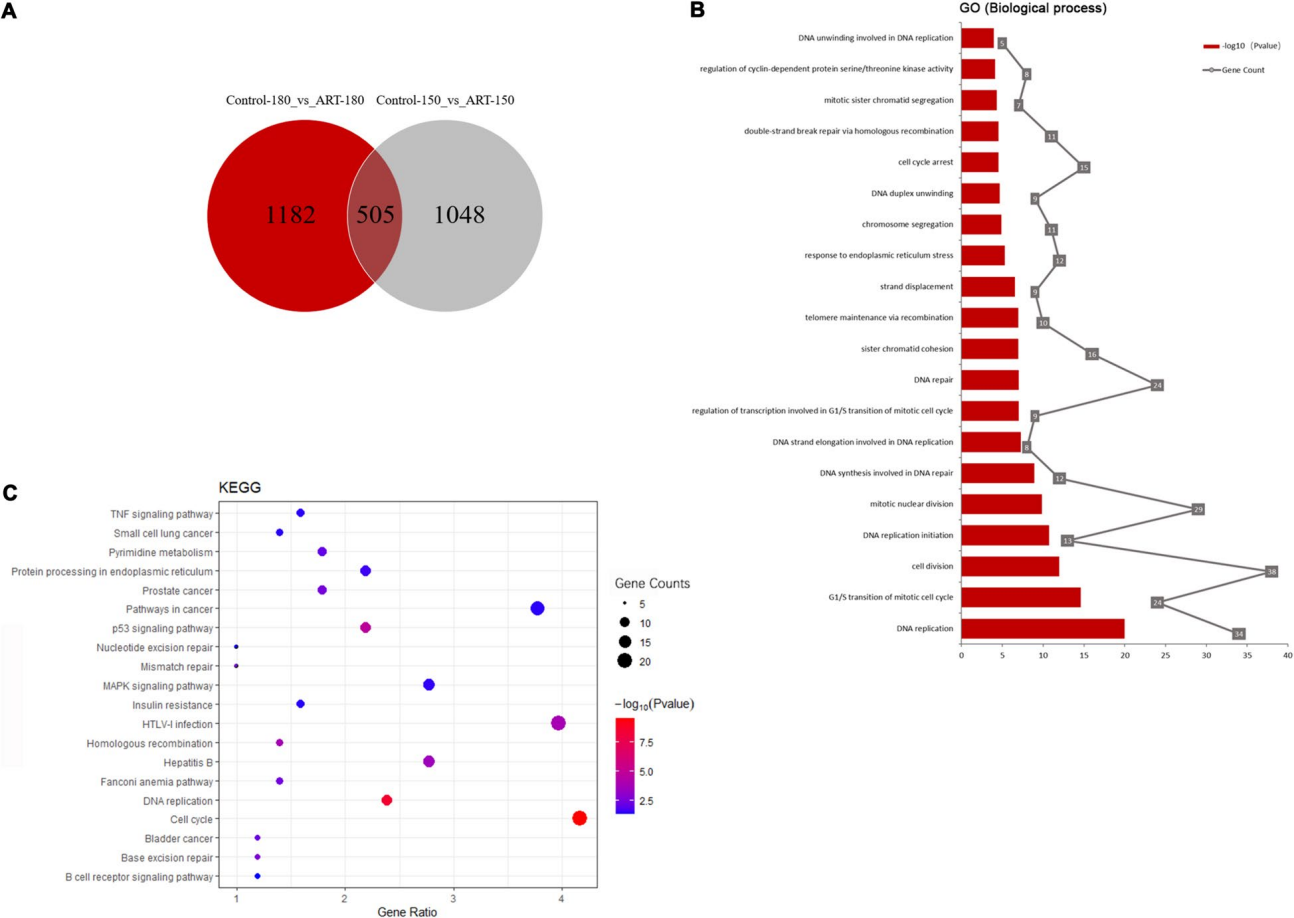
Changes in gene expression in ESCC cells treated with ART. **A** Venn diagram of DEGs in KYSE180 and KYSE150 cells untreated or treated with 50 μM ART for 48 h. EBSeq software was used for differential gene expression analysis, and fold change ≥ 2 or ≤ 1/2 and FDR < 0.01 were used as the screening standards. **B** KEGG analysis of 505 DEGs detected in both the KYSE180 and KYSE150 cell lines treated with ART. **C** GO analysis of 505 DEGs detected in both the KYSE180 and KYSE150 cell lines treated with ART

To investigate the mechanism(s) by which ART induces cell cycle arrest/apoptosis in ESCC cells, we performed RNA transcriptomic profiling of KYSE150 and KYSE180 cells treated with or without ART using RNA sequencing (RNA-seq). As shown in Fig. 5A, RNA-seq indicated that, compared with the controls, ART treatment significantly altered 1182 genes in KYSE150 cells, whereas ART treatment markedly altered 1048 gene expressions in KYSE180 cells. We identified 505 differentially expressed genes that could be detected in both ESCC cell lines treated with ART (Fig. 5A). KEGG and GO analyses of these 505 genes revealed that they were mainly involved in regulating cell cycle progression, especially G1 progression (*CCNE2*, *CCNA2*, *CDKN1A*, etc.); DNA metabolism, such as DNA replication, recombination, repair and S-phase checkpoint control (*MCM2*, *MCM3, XRCC2*, etc.); and p53 signalling and signal transduction pathways (*TNF*, *MAPK*, etc.) (Fig. 5B, C). These results indicated that ART treatment in ESCC cells, which induces ROS, cellular oxidative stress and DNA damage, triggered cell cycle checkpoint and/or DNA damage responses, ultimately resulting in cell cycle arrest/apoptosis.

**Fig. 6:**
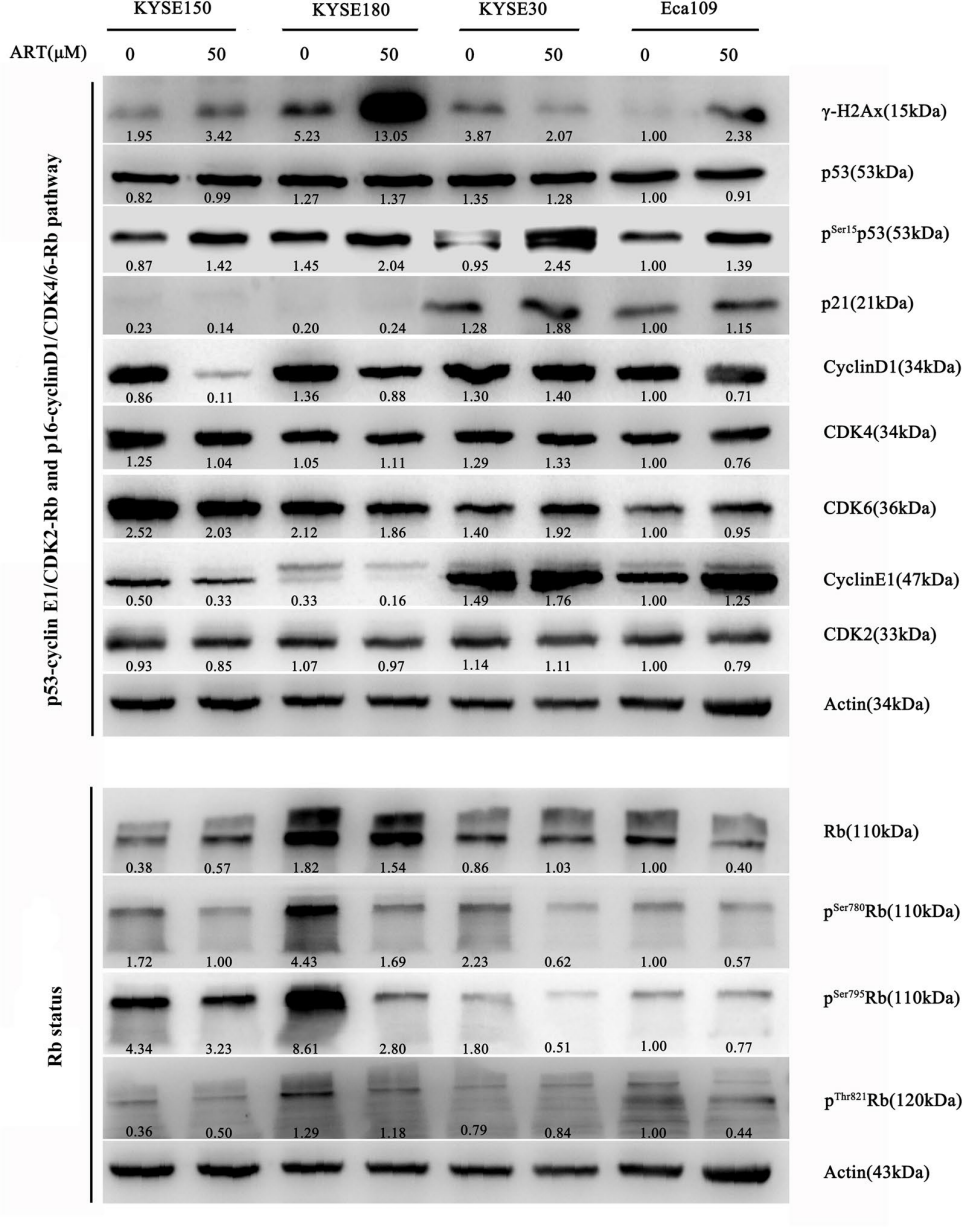
Western blot analyses were performed for proteins related to the cyclin/CDK-Rb signalling pathway and the status of phosphorylated Rb in different ESCC cell lines after treatment with 50 μM ART for 24 h

We assessed how ART induced cell cycle and/or DNA damage checkpoint responses in ESCC cells in detail. Immunoblotting indicated that ART treatment of KYSE150, KYSE180 and Eca109 cells resulted in increased γ-H2AX levels (Fig. 6). Although immunofluorescence also demonstrated that ART treatment caused increased γ-H2AX levels in KYSE30 cells (Fig. 1C), increased γ-H2AX levels were not detected by immunoblotting mainly because ART induced massive cell death in these cells (Fig. 2A, B). Thus, these results indicated that ART-generated ROS cause oxidative DNA damage that also triggers the ATM/ATR-Chk2/Chk1-γ-H2AX DNA damage response in ATR-treated ESCC cells.

Given that ART-induced cell cycle arrest and apoptosis in ESCC cells are cell line dependent, we asked whether genomic alterations could impact the effects of ATR in these cell lines. Analysis of the Cancer Cell Line Encyclopedia revealed that KYSE30 cells harbouring *CDKN2A* gene mutations had wild-type *TP53* (*p53*^*Nor*^*/p16*^*Mut*^), whereas KYSE150 and KYSE180 cells harbouring *TP53* gene mutations had wild-type *CDKN2A* (*p53*^*Mut*^*/p16*^*Nor*^). In contrast, Eca109 had both wild-type *TP53* and *CDKN2A* genes (*p53*^*Nor*^*/p16*^*Nor*^) (Table 1). It is possible that p53-dependent and/or Cdk4/6-p16-Rb-dependent checkpoint controls play crucial roles in mediating ART cytotoxicity in ESCC cell lines.

To test this possibility, we assessed the p53-dependent checkpoint response in these four ESCC cell lines treated with ART. Consistent with their *TP53* genotypes, immunoblotting showed that ATR-treated KYSE30 and Eca109 cells with wild-type *TP53* genes displayed increased p53 phospho-Ser15 levels and p53 downstream target p21 expression, whereas ART-treated KYSE150 and KYSE180 cells with mutated *TP53* genes did not show these effects compared with controls (Fig. 6). Next, we assessed the Cdk4/6-p16-Rb-dependent checkpoint response in these four ESCC cell lines treated with ART. Although the interplay between the p53 and Cdk-Rb pathways could be complex, immunoblotting revealed that ART treatment of KYSE150 and KYSE180 cells (*p53*^*Mut*^*/p16*^*Nor*^) resulted in decreased cyclin D1 expression and hypo-phosphorylated Rb levels compared with those in controls. In contrast, ART treatment in KYSE30 cells (*p53*^*Nor*^*/p16*^*Mut*^) did not affect cyclin D1 or Cdk4/6 expression, thus resulting in increased hyperphosphorylated Rb levels compared with those in controls. ART treatment of Eca109 cells (*p53*^*Nor*^*/p16*^*Nor*^) also had minimal effects on cyclin D1/Cdk4 expression compared with the control. However, Eca109 cells treated with ART displayed increased levels of hypo-phosphorylated Rb, consistent with the finding that ATR treatment only induced cell cycle arrest in this cell line. These results, together with the cell viability analysis presented above (Fig. 1D), indicated that p53-dependent and/or Cdk4/6-p16-Rb-dependent checkpoint controls played critical roles in mediating ART cytotoxicity in ESCC cell lines.

### Manipulations of p53 and Cdk4/6-p16-Rb activities altered ART cytotoxicity in ESCC cells

Based on the results we obtained, we therefore hypothesized that the strong discrepant cytotoxic effects of ART among ESCC cell lines were due to the synergistic effects of ART-dependent cellular oxidative stresses such as extensive DNA damage and cell cycle checkpoint regulation under specific genomic backgrounds. We postulated that in KYSE30 cells (*p53*^*Nor*^*/p16*^*Mut*^) treated with ART, p16 deficiency would result in superactive Cdk4/6, leading to defects in Cdk4/6-p16-Rb checkpoint control and powerful G1 progression, whereas normal p53 would allow ART-induced cellular oxidative stresses activating p53-dependent cell cycle checkpoint control to maintain the cell cycle in the late G1 phase. These paradoxical regulatory effects ultimately resulted in cell collapse and death in the G1/S-phase such that KYSE-30 cells were very sensitive to ART treatment. In contrast, because both the p53 and Cdk4/6-p16-Rb cell cycle checkpoints were intact in Eca109 cells (*p53*^*Nor*^*/p16*^*Nor*^) treated with ART, their activation resulted in cell G1 arrest. Hence, Eca109 cells were more resistant to ART treatment.

**Fig. 7:**
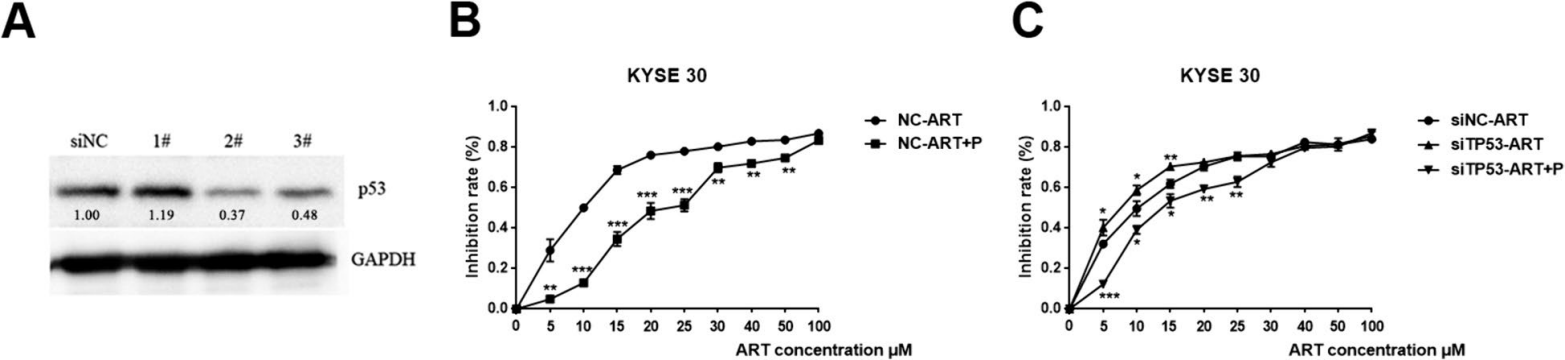
Different *CDKN2A* and *TP53* mutation statuses determine cell sensitivity to ART. **A** Western blot analyses were performed for p53 in siNC KYSE30 cells and three *TP53* siRNA KYSE30 cell lines, and #2 was selected. **B** The effects of treatment with the Cdk4/6-specific inhibitor palbociclib on the viability of KYSE30 cells treated with different concentrations of ART, as detected using the CCK-8 assay. The inhibition rates are the ratios of the number of dead cells treated with ART to the number of living cells not treated with ART. Palbociclib significantly rescued the tolerance of KYSE30 cells to ART. **C** The effects of treatment with the Cdk4/6-specific inhibitor palbociclib on the viability of cells treated with different concentrations of ART, as detected using the CCK-8 assay. *TP53* silencing increased the sensitivity of KYSE30 cells to ART, whereas palbociclib increased the tolerance of these cells. *n* = 3, *P < 0.05, **P < 0.01, ***P < 0.001

To verify our hypothesis, we manipulated Cdk4/6-p16-Rb activity using the Cdk4/6-specific inhibitor palbociclib and/or p53 function using *TP53* siRNA in KYSE30 cells treated with ART (Fig. 7A). Palbociclib significantly increased the tolerance of KYSE30 cells to ART, with an IC50 value of 33 μM, compared with that of KYSE30 cells treated with ART alone at 11 μM (Fig. 7B, Table 2). In contrast, when *TP53* was silenced, KYSE30 cells became more sensitive to ART, with an IC50 value of 8 μM. However, the addition of palbociclib to p53-ablated KYSE30 cells restored the IC50 of ART to 22 μM. Consistently, treatment with palbociclib also partially restored the viability of p53-ablated KYSE30 cells (Additional file 3). The combined treatments of palbociclib and *TP53* siRNA in ART-treated KYSE30 cells partially mimicked ART treatments in KYSE150 and KYSE180 cells (*p53*^*Mut*^*/p16*^*Nor*^), which had higher IC50 values for ART than KYSE30 cells (Fig. 7C, Table 2). Hence, these results showed that ART cytotoxicity was closely related to the genomic status of p53 and Cdk4/6-p16-Rb, indicating that ART is potentially useful for neoadjuvant chemotherapy against ESCC.

**Table 2:** IC50s of KYSE30 treated with ART under different conditions

Cell lines	IC50
NC + ART	10.87 ± 0.92 μM
NC + ART + P	33.27 ± 1.27 μM
siNC + ART	12.77 ± 0.45 μM
siTP53 + ART	7.66 ± 0.43 μM
siTP53 + ART + P	22.37 ± 0.33 μM

P stands for palbociclib

## Discussion

Previous studies revealed the profound cytotoxic effects of the antimalarial drug ART, which generates ROS following the breakage of its endoperoxide bridge, resulting in intracellular oxidative stress in various types of cancer cells [11]. However, ESCC cells have rarely been studied, and the detailed underlying mechanisms by which ART induces cancer cell death remain elusive. We examined the antitumour effects of ART in ESCC cells and showed that, similar to other tumour cell lines treated with ART, ART-treated ESCC cell lines produced excessive ROS, resulting in DNA damage, cell cycle arrest and/or apoptosis. ART treatment of ESCC cell lines also inhibited clonogenicity and tumorigenicity both in vitro and in vivo. Furthermore, we revealed that ART cytotoxicity in ESCC cells was cell line dependent and mediated by p53 and Cdk4/6-p16-Rb cell cycle checkpoint controls. Alterations in p53 and the Cdk4/6-p16-Rb cell cycle checkpoint control perturbed ATR cytotoxicity in ESCC cells.

A large body of evidence from omic data has demonstrated that genes involved in the p53 and Cdk4/6-cyclin D1-p16-Rb cell cycle checkpoint pathways are frequently mutated in ESCCs [31]. In their 2017 whole-genome sequencing of DNA and RNA in 94 Chinese individuals with ESCC, Chang et al. reported an extremely high frequency of *TP53* mutations (85%) and statistically significant single nucleotide variations in several other genes, including *CDKN2A* (7%) [4]. Cui et al. performed deep whole-genome sequencing on ESCC samples from 508 individuals and identified 22 candidate driver genes. All 22 of these genes had moderate to high expression in ESCC, including *TP53* (75%), *CCND1* (35%), *CDKN2A* (31%), *CDKN2B* (20%), *CDK6* (8%), *E2F1* (6%), *Rb* (5%) and *CDK4* (2%), suggesting that variations in p53 and Cdk4/6-p16-Rb cell cycle checkpoint controls are critical events in ESCC [8]. Given that ESCC cell lines show different sensitivities to ART treatment, four ESCC cell lines with different genomic backgrounds of p53 and Cdk4/6-p16-Rb cell cycle checkpoint pathways were utilized for detailed analyses of the effects of ART on ESCC cells. Our results demonstrated that KYSE150 and KYSE180 cells, which are deficient in the p53 checkpoint pathway but are proficient in the Cdk4/6-p16-Rb checkpoint pathway, displayed moderate sensitivities to ART treatment. In contrast, KYSE30 cells, which are deficient in the Cdk4/6-p16-Rb checkpoint pathway but are proficient in the p53 checkpoint pathway, showed high sensitivity to ART treatment. In contrast, Eca109, with intact p53 and Cdk4/6-p16-Rb cell cycle checkpoint control pathways, exhibited high resistance to ART. These results indicated the importance of the status and interplay between p53 and the Cdk4/6-p16-Rb cell cycle checkpoint control pathways in controlling cell tolerance to ART.

Consistently, KYSE150 and KYSE180 cells treated with ART exhibited reduced cyclin D1 expression, thus reducing the activity of the cyclin D1-CDK4/6 complex. Rb was hypo-phosphorylated, resulting in G1/S arrest. However, these cells with p53 deficiency might not be able to repair DNA damage induced by ART and ultimately undergo apoptosis. In contrast, ART treatment of KYSE30 cells did not affect the Cdk4/6-p16-Rb checkpoint control pathway because of p16 deficiency. ART-treated cells progressed through the G1 to S-phase as cyclin D1-Cdk4/6 was overactivated. However, these cells also activated the p53 cell cycle checkpoint pathway via ART-dependent cellular oxidation and DNA damage, attempting to arrest cells at G1/S. The paradoxical effects on the p53 and Cdk4/6-p16-Rb checkpoint control pathways ultimately led to cell collapse and death. Thus, these results indicated that ESCC cells with an intact p53 checkpoint control pathway but a defective Cdk4/6-p16-Rb checkpoint control pathway were most sensitive to ART treatment [18, 32]. Conversely, Eca109 cells that retained both the intact p53 and Cdk4/6-p16-Rb cell cycle checkpoint control pathways arrested cells at G1/S after ART treatment, thus showing ART resistance. Overall, our results demonstrated that dysfunctions in the p53 and Cdk4/6-p16-Rb cell cycle checkpoint control pathways, especially the Cdk4/6-p16-Rb cell cycle checkpoint control pathway, increase the vulnerability of ESCC cells to ART treatment.

Indeed, manipulations of p53 and/or Cdk4/6-p16-Rb cell cycle checkpoint controls strongly affected ATR cytotoxicity in ESCC cells. We showed that palbociclib, a specific CDK4/6 inhibitor, reduces ART toxicity in KYSE30 cells. A possible explanation is that, together with the normal p53 checkpoint control, palbociclib treatment of KYSE30 cells restored Cdk4/6-p16-Rb cell cycle checkpoint control, blocked cell cycle progression and contributed to resistance to ART treatment. Given that the abundant ROS-induced toxicity induced by ART was similar to the effects of ionizing radiation and cytotoxic chemotherapies, such as Adriamycin and platinum drugs, our study further indicated that palbociclib may not demonstrate efficacy when used concurrently with DNA damage therapies. Consistent with our study, Fei’s study indicated that ART enhanced the radiosensitivity of oesophageal cancer cells by inhibiting DNA damage repair processes. ART induces ESCC cell arrest at the G2/M phase; attenuates the effects of Ku70, Ku86, RAD51, and RAD54 protein activation, which contributes to DNA DSB damage repair; and sensitizes EC cells to radiation [12]. Therefore, appropriate treatments for ESCCs should be formulated according to the genomic background and combination therapy. For example, for ESCCs with mutations in the *TP53* gene but a normal Cdk4/6-cyclin D1-p16-Rb pathway, similar to that noted in KYSE150 and KYSE180 cells, ART and/or DNA-damaging therapies could demonstrate efficacy [17, 20, 27]. However, neither the Cdk inhibitor palbociclib nor DNA-damaging drugs should be used for the treatment of ESCCs with a normal *TP53* gene but an abnormal Cdk4/6-cyclin D1-p16-Rb pathway, similar to that noted in KYSE30 cells. Precision therapies for ESCCs using multimodality medicines should be considered cautiously rather than as causal combinations, depending on tumour genetics.

## Conclusions

This study showed that ART inhibits the growth of ESCC cells in vitro and the tumorigenicity of ESCC cells in vivo. ART exerted its anticancer effects by inducing ROS production and DNA oxidative damage, thus activating the p53/Cdk4/6-p16-Rb cell cycle checkpoint control pathways in ESCC cells. Given that the p53 and Cdk4/6-cyclin D1-p16-Rb genes are commonly mutated in ESCC, it was hypothesized that ART is potentially clinically useful as a chemotherapeutic or adjuvant drug for ESCC.

### Supplementary Information

Supplementary material 1.

Supplementary material 2.

Supplementary material 3.

Supplementary material 4.

## Data Availability

The datasets generated and/or analysed during the current study are available in the GSA database repository [https://ngdc.cncb.ac.cn/gsa-human/browse/HRA002413].

## Abbreviations

EC: Oesophageal cancer
EAC: Oesophageal adenocarcinoma
ESCC: Oesophageal squamous cell carcinoma
ART: Artemisinin
ROS: Reactive oxygen species
NAC: N-Acetyl-L-cysteine
STR: Short tandem repeat
IC50: Half inhibitory concentration
FACS: Fluorescence activated cell sorting
