# Supplementary material for: Antitumour effects of artesunate via cell cycle checkpoint controls in human oesophageal squamous carcinoma cells
Source: Eur J Med Res. 2024 May 22;29:293. doi: 10.1186/s40001-024-01882-9 (PMC11110347; doi:10.1186/s40001-024-01882-9)
Supplement: Supplementary file 1 — Supplementary material 1. [file 40001_2024_1882_MOESM1_ESM.docx]

**Additional files**

Additional file 1. The effects of different concentrations of ART on apoptosis of different ESCC cell lines. Cells were treated with 25μM and 50μM ART for 24 and 48 hours, and the apoptotic cells were stained by Annexin V-FITC and propidium iodide (PI). The lower left quadrants (FITC-/PI-) indicated the viable cells, and the lower right quadrants (FITC+/PI-) indicated the early apoptotic cells. The upper right quadrants (FITC+/PI+) indicated the late apoptotic cells, and the upper left quadrants (FITC-/PI+) indicated the dead cells.

Additional file 2. The effects of different concentrations of ART on the cell cycle of different ESCC cell lines. Cells were treated with 25μM and 50μM ART for 24 and 48 hours, and DNA ploidy was assessed by PI staining and flow cytometry.

Additional file 3. The effects of Cdk4/6 specific inhibitor Palbociclib treatment on viability of KYSE30 cells detected by CCK-8. The inhibition rates are ratios of the number of dead cells treated with Palbociclib to the number of living cells untreated with Palbociclib. *P < 0.05, **P <0 .01.
