# Supplementary figures and images for: Antitumour effects of artesunate via cell cycle checkpoint controls in human oesophageal squamous carcinoma cells
Source: Eur J Med Res. 2024 May 22;29:293. doi: 10.1186/s40001-024-01882-9 (PMC11110347; doi:10.1186/s40001-024-01882-9)

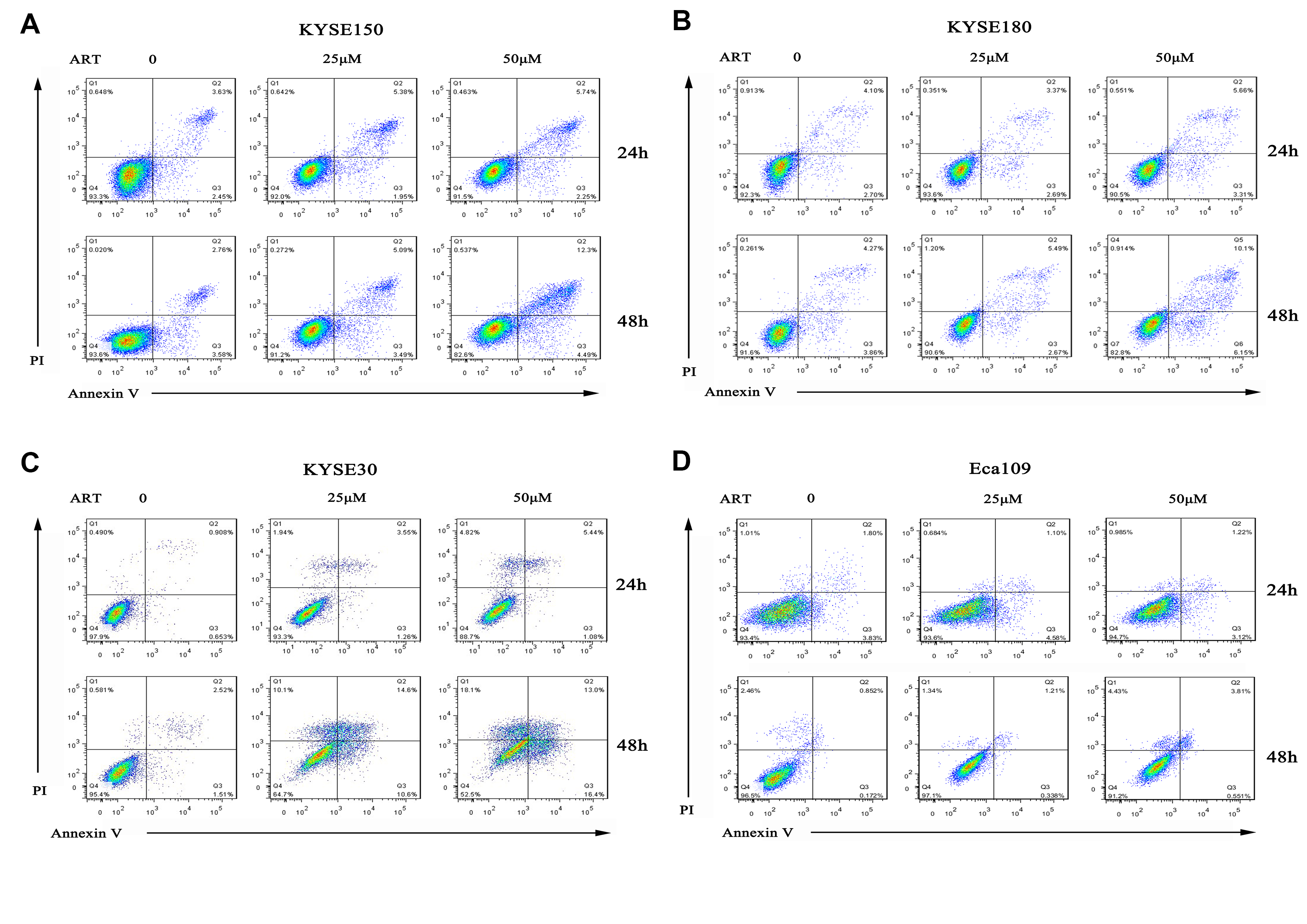

Supplement: Supplementary file 2 — Supplementary material 2. [file 40001_2024_1882_MOESM2_ESM.png]

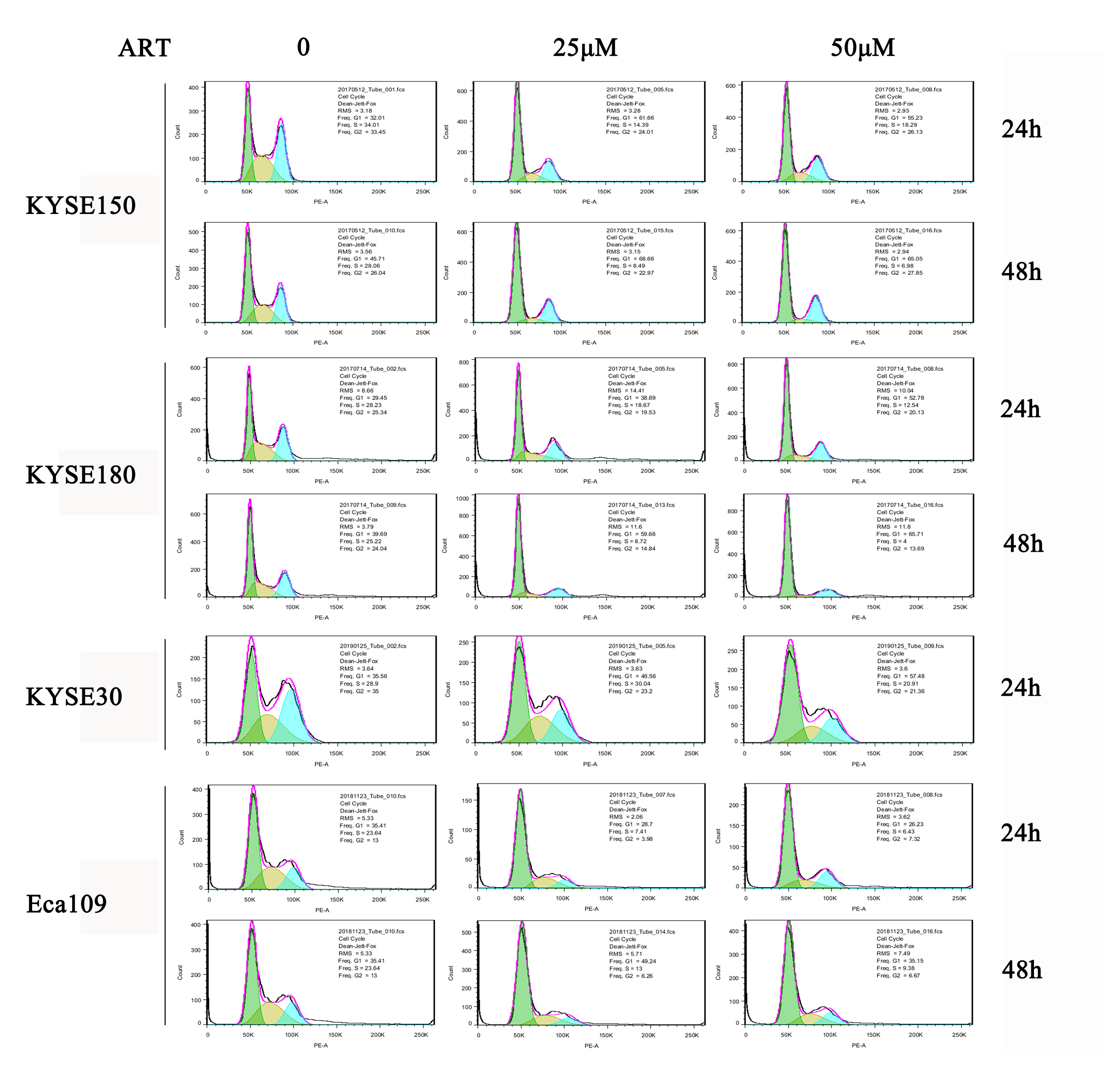

Supplement: Supplementary file 3 — Supplementary material 3. [file 40001_2024_1882_MOESM3_ESM.png]

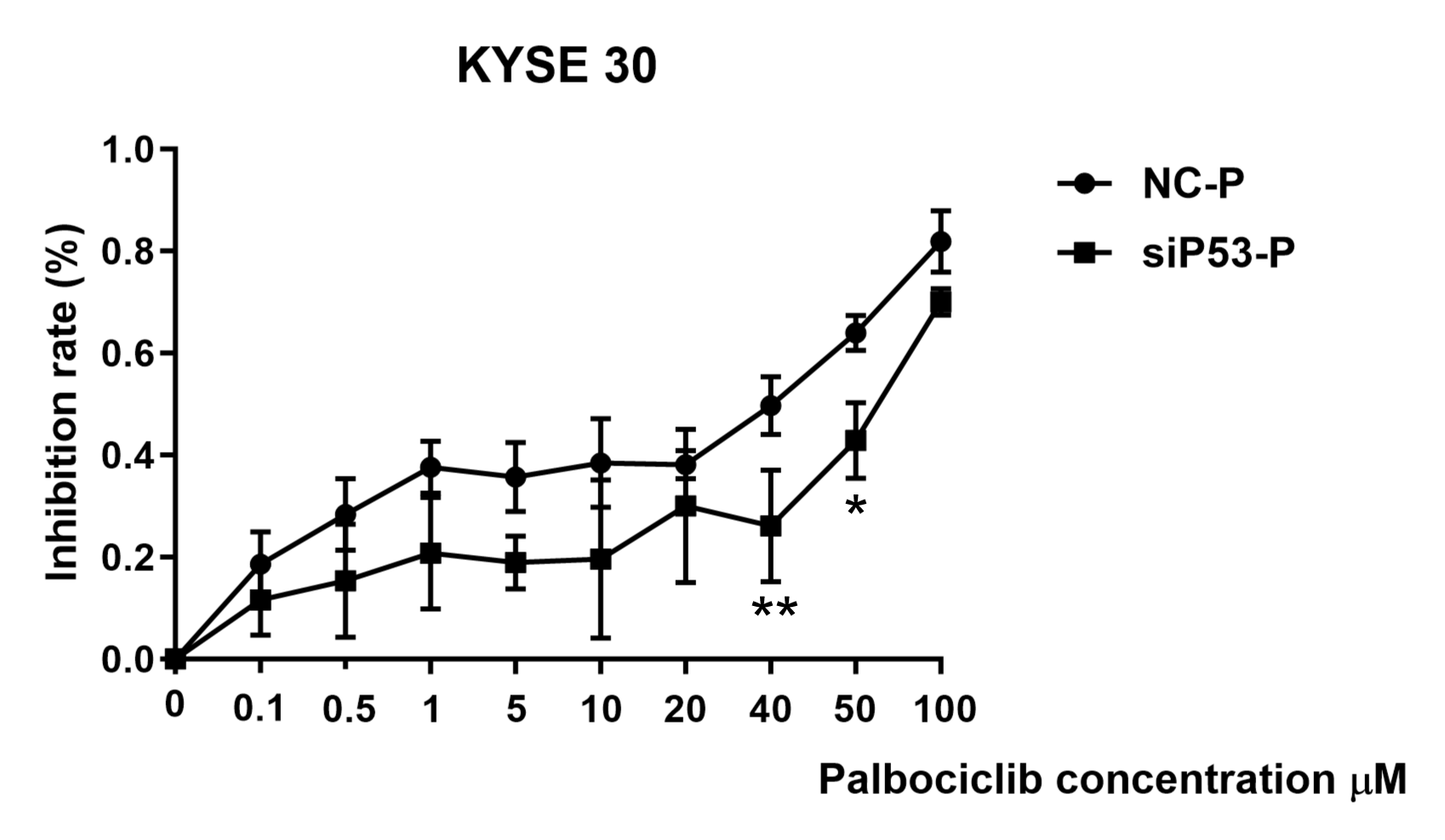

Supplement: Supplementary file 4 — Supplementary material 4. [file 40001_2024_1882_MOESM4_ESM.png]
